# A Customized Screening Tool Approach for the Development of a Self-Nanoemulsifying Drug Delivery System (SNEDDS)

**DOI:** 10.1208/s12249-021-02176-7

**Published:** 2021-12-27

**Authors:** Fabian-Pascal Schmied, Alexander Bernhardt, Andrea Engel, Sandra Klein

**Affiliations:** 1 Department of Pharmacy, Institute of Biopharmaceutics and Pharmaceutical Technology, University of Greifswald, Felix-Hausdorff-Straße 3, 17489 Greifswald, Germany; 2 Research, Development & Innovation, Evonik Operations GmbH, Kirschenallee, 64293 Darmstadt, Germany

**Keywords:** solubility enhancement, triangular mixture design, nanoemulsion, drug release, dynamic light scattering

## Abstract

The present study focused on establishing a novel, (pre-)screening approach that enables the development of promising performing self-nanoemulsifying drug delivery systems (SNEDDSs) with a limited number of experiments. The strategic approach was based on first identifying appropriate excipients (oils/lipids, surfactants, and co-solvents) providing a high saturation solubility for lipophilic model compounds with poor aqueous solubility. Excipients meeting these requirements were selected for SNEDDS development, and a special triangular mixture design was applied for determining excipient ratios for the SNEDDS formulations. Celecoxib and fenofibrate were used as model drugs. Formulations were studied applying a specific combination of *in vitro* characterization methods. Specifications for a promising SNEDDS formulation were self-imposed: a very small droplet size (< 50 nm), a narrow size distribution of these droplets (*PDI* < 0.15) and a high transmittance following SNEDDS dispersion in water (> 99% in comparison with purified water). Excipients that provided a nanoemulsion after dispersion were combined, and ratios were optimized using a customized mapping method in a triangular mixture design. The best performing formulations were finally studied for their *in vitro* release performance. Results of the study demonstrate the efficiency of the customized screening tool approach. Since it enables successful SNEDDS development in a short time with manageable resources, this novel screening tool approach could play an important role in future SNEDDS development.

**Figure:**
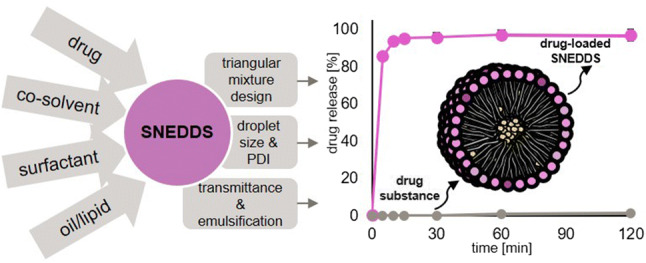
Graphical abstract

Graphical abstract

## INTRODUCTION

The majority of new drug candidates in current research and development pipelines are associated with poor aqueous solubility. This may lead to low bioavailability (1–3) and is thus one of the main limitations for oral drug delivery. According to recent reports, currently, about 40% of the drugs on the market and about 75% of the drugs in the state of development are poorly water-soluble (4). Drugs with poor aqueous solubility can be further distinguished into “grease ball” and “brick dust” molecules. The aqueous solubility of “grease ball” molecules is substantially limited by their pronounced lipophilic character (Log*P* > 3), while “brick dust” molecules are characterized by very high melting points (*T*_*m*_ > 200 °C) resulting in a high crystal lattice energy that severely impedes the drugs’ solubility behavior (5, 6). In view of this growing number of poorly soluble drug candidates, it is proving necessary to establish formulation approaches to counteract the solubility issues of these drugs. Lipid-based drug delivery systems possess the ability to address an inadequate aqueous solubility (7). Especially for “grease ball” molecules, numerous studies applying lipid-based drug delivery systems have been published, while corresponding studies on “brick dust” molecules are sort of lacking (5). One of the lipid-based drug delivery systems that address the challenge of solubility enhancement of poorly water-soluble drugs is referred to as self-nanoemulsifying drug delivery system (SNEDDS) (2, 8). SNEDDSs are multicomponent, homogeneous, anhydrous liquids that, after oral administration, under the mild agitation of digestive motility, spontaneously form translucent emulsions upon contact with gastrointestinal fluids (3, 9). They consist of an oil or a lipid that is combined with a surfactant or a blend of surfactants, a co-solvent, if required, and can incorporate a lipophilic drug (3, 8, 10–12). The variety of components that can be used for SNEDDS reveals the complexity of these systems and the almost endless number of possible combinations with each other (13). On the one hand, this innumerable combination variety tremendously increases the possibilities to develop a functioning SNEDDS formulation. On the other hand, the high number of conceivable combinations also requires a lot of time and experimental effort when it comes to finding the right formulation approach. It would therefore be desirable to develop a screening tool that allows successful formulation approaches to be determined in a short time and with manageable experimental effort. The screening tool should target on selecting those SNEDDS components that in an appropriate mixing ratio provide excellent emulsification properties (*i.e.*, the formation of a nanoemulsion) for the drug compound of interest. Dissolving the drug in a water-free preconcentrate and obtaining a stable nanoemulsion after dispersion in an aqueous medium avoids the dissolution step of the solid prior to drug absorption and thus can facilitate drug absorption in the intestine. Particularly for highly permeable drug compounds, this can present with a higher bioavailability, which in this case is also often accompanied by more reliable *in vivo* plasma levels (14, 15).

Since a SNEDDS formulation is intended to solubilize a drug with poor aqueous solubility in the contents of the gastrointestinal tract, solubility of the drug both in the individual components of the SNEDDS as well as in the complex mixture of these components is a fundamental requirement. It is essential that the individual components of SNEDDS are also miscible with each other. As a first step in determining an appropriate mixture of constituents for a SNEDDS formulation, the saturation solubility of a lipophilic drug in the individual components that would generally be suitable for SNEDDS development should be investigated (3, 7, 9, 16–20). Components that present with a high saturation solubility for the drug of interest are promising candidates for the application in mixtures for SNEDDS (8, 16). However, the successful use of an excipient in a SNEDDS formulation requires the compatibility of all individual constituents with each other (8, 16). Moreover, as stated before, to ensure proper dispersion and to prevent drug precipitation in gastrointestinal fluids, SNEDDS should provide stable and transparent nanoemulsions after dispersion. Usually, ternary phase diagrams are constructed to determine the self-emulsifying region of a certain excipient mixture, and if a compatible excipient mixture enabling a high drug loading has been determined, a number of characterization methods such as droplet size analysis and transmittance measurement of SNEDDS after dispersion in water are applied to determine an appropriate mixing ratio of the components including the drug (20–25).

The conventional way of optimizing a ternary mixture requires about 50 individual experiments since the composition is usually varied in 10% increments to determine the optimal SNEDDS formulation.

A novel, customized screening approach distinct from the 10% increment procedure, might reduce the number of trials for developing SNEDDS formulations and could thus help to streamline the screening process for SNEDDS development. The aim of the present study was thus to establish a novel and tailored screening tool approach for the initial, rapid development of promising SNEDDS formulations which should be based on the triangular mixture design and provide SNEDDS mixtures that fit with a set of self-imposed specifications such as providing very small nanodroplets (< 50 nm) that are narrowly distributed (polydispersity index (PDI) < 0.15) and provide a highly translucent (> 99%), stable nanoemulsion after dispersion in aqueous fluids. If these requirements are met, the resulting SNEDDS formulations should lead to improved solubility and higher dissolution rates of the investigated active pharmaceutical ingredients (APIs) and also ensure long-term stability of the formulation.

## MATERIALS AND METHODS

### Materials

Celecoxib and fenofibrate were used as model drug substances. Celecoxib was obtained from Aarti Drugs Ltd. (Mumbai, India) and fenofibrate from D.K. Pharma Chem PVT Ltd. (Maharashtra, India). Polyoxyethylene (80) sorbitan monooleate (Tween^®^ 80), d-α-tocopherol polyethylene glycol 1000 succinate (d-TPGS, Tocophersolan), isopropyl myristate (IPM-100), polyoxyl-(23) lauryl ether (Brij^®^ 35), castor oil, oleic acid, corn oil, olive oil, peanut oil, soybean oil, polyoxyl-40 hydrogenated castor oil (Cremophor^®^ RH 40), sorbitan sesquioleate (Span^®^ 83), polyoxyethylene sorbitan monolaurate (Tween^®^ 20), and tetraethylene glycol (Tetra EG) were purchased from Sigma Aldrich (Steinheim, Germany). Lauroyl polyoxyl-32 glycerides (Gelucire^®^ 44/14), oleoyl polyoxyl-6 glycerides (Labrafil^®^ M 1944 CS), linoleoyl polyoxyl-6 glycerides (Labrafil^®^ M 2125 CS), caprylocaproyl polyoxyl-8 glycerides (Labrasol^®^), propylene glycol monolaurate (type II) (lauroglycol™ 90), propylene glycol monolaurate (type I) (Lauroglycol™ FCC), glyceryl monolinoleate (Maisine™ CC), glyceryl monooleate (Peceol™), polyglyceryl-3 dioleate (Plurol^®^ Oleique CC 497), glyceryl tricaprylate/tricaprate (Labrafac™ lipophile WL 1349), propylene glycol dicaprylate/dicaprate (Labrafac™ PG) and diethylene glycol monoethyl ether (Transcutol^®^ HP) were kindly donated by Gattefossé S.A.S (Saint Priest, France). Medium-chain triglycerides (Miglyol^®^ 812) and polyethylene glycol 400 were obtained from Caesar & Loretz GmbH (Hilden, Germany). Ethyl oleate (Crodamol™ EO), sorbitan monolaurate (Span^®^ 20), sorbitan monooleate (Span^®^ 80) and propane-1,2,3-triol were provided by Merck KGaA (Darmstadt, Germany). Polyoxyl-35 hydrogenated castor oil (Kolliphor^®^ EL) and polyoxyl-15 hydroxystearate (Kolliphor^®^ HS 15) were obtained from BASF SE (Ludwigshafen, Germany). Propylene glycol was purchased from VWR International GmbH (Darmstadt, Germany). Fish oil (EPA/DHA) is an in-house product of Evonik Operations GmbH (Hanau, Germany). All other chemicals and solvents were of analytical grade and purchased commercially.

### HPLC Equipment for Analyzing Drug Substances

A high-performance liquid chromatography (HPLC) system (Agilent 1260 Infinity) was used for the quantification of the model drug substances. The system consisted of a quaternary pump (G1311B), autosampler (G1329B), column oven (G1316A), and UV detector (G1314C), all from Agilent Technologies (Frankfurt am Main, Germany). The validation of the applied analytical methods was conducted according to USP specifications.

### HPLC Method for Analyzing Celecoxib

Separation of all samples containing celecoxib was achieved using a Knauer Nucleosil 100–7 C18 (125 × 4.6 mm, 7 µm) column maintained at 40 °C. The mobile phase consisted of an acetonitrile:water:triethylamine mixture (300:300:0.9 v/v), adjusted to pH 3.00 with phosphoric acid. The flow rate was set to 1.8 ml/min. An injection volume of 5 µl was applied, and celecoxib was detected at 254 nm. In the concentration range of 0.13–542 µg/ml, the analytical curve was linear (*r*^2^ = 0.999995). The method was found to be accurate (100.2–102.1%) and precise (CV 2.46%) with a quantification limit of 0.05 µg/ml. Run time was defined as 7 min.

### HPLC Method for Analyzing Fenofibrate

Separation of all samples containing fenofibrate was achieved using a Symmetry 300 C18 (150 × 4.6 mm, 5 µm) column maintained at 22 °C. The mobile phase consisted of an acetonitrile:water mixture (70:30 v/v), adjusted to pH 2.50 with phosphoric acid. The flow rate was set to 2.0 ml/min. An injection volume of 20 µl was applied, and fenofibrate was detected at 286 nm. In the concentration range of 0.13–526 µg/ml, the analytical curve was linear (*r*^2^ = 0.999992). The method was found to be accurate (101.2–101.4%) and precise (CV 2.42%) with a quantification limit of 0.05 µg/ml. Run time was defined as 6 min.

For both HPLC methods, the selectivity for the respective drug substances was determined (in the presence of each of the excipients used in the formulations). No interference was observed in drug retention time, and the peak area did not change.

### Solubility Studies of Celecoxib and Fenofibrate in Various Excipients

The saturation solubility of each drug in each of the individual excipients was investigated in triplicate by the following procedure: first, drug and excipients were blended in 2-ml safe lock test tubes (17, 23, 26). After short, intense vortex mixing, the mixtures were agitated at 1000 rpm for 24 h at 37 °C under light protection using a ThermoMixer^®^ C (Eppendorf AG, Hamburg, Germany) to achieve an equilibrium state (7, 12, 16, 17, 23, 26, 27). Subsequently, the samples were centrifuged for 1 min at 9300 relative centrifugation forces (rcf) in a preheated (37 °C) centrifuge to remove drug substance that had not dissolved (16, 17, 19, 26–28). After centrifugation at 37 °C, a defined mass (50 mg) of the supernatant was withdrawn and transferred to a 25-ml volumetric flask, and the sample was diluted with methanol to a total volume of 25.0 ml. Finally, a sample of 1 ml was analyzed using one of the previously described drug-specific HPLC methods.

### Excipient Compatibility Assessment

Following the solubility studies, the excipients were tested for their miscibility or compatibility. For this purpose, binary mixtures in different mixing ratios were prepared in each case and visually inspected (data not shown). The miscibility of the individual excipients as well as the solubility of the specific drug in the individual excipients were considered as decisive criteria for the compatibility evaluation of promising excipient candidates for further SNEDDS production. Consequently, only excipients that successfully passed the compatibility test (*i.e.*, resulted in a single-phase stable system without detectable droplets, particles, or even complete phase separation) were used in the following experiments.

### Triangular Mixture Design

Once a mixture of compatible excipients was identified for a given drug compound, in the presence of the API of interest, a systematic optimization of the excipient mixing ratio was performed with respect to the set specifications, namely a droplet size < 50 nm, a PDI < 0.15, and a transmittance > 99% after dispersing the SNEDDS formulation in water. For this purpose, a triangular mixture design, in which the three individual components must sum up to 100% was applied. Prior to assessing the emulsion performance, celecoxib or fenofibrate was dissolved in each individual SNEDDS mixture prepared in the screening experiments. The drug load applied in these experiments was calculated based on API solubility in the individual components of the SNEDDS mixture using the following equation:

1 $$ Drug\kern0.277778em load\kern0.277778em \left[\%\right]=f\ast \frac{\sum_{i=1}^n{\left({c}_s\right)}_i}{n}\ast 0.1 $$

**Fig. 1:**
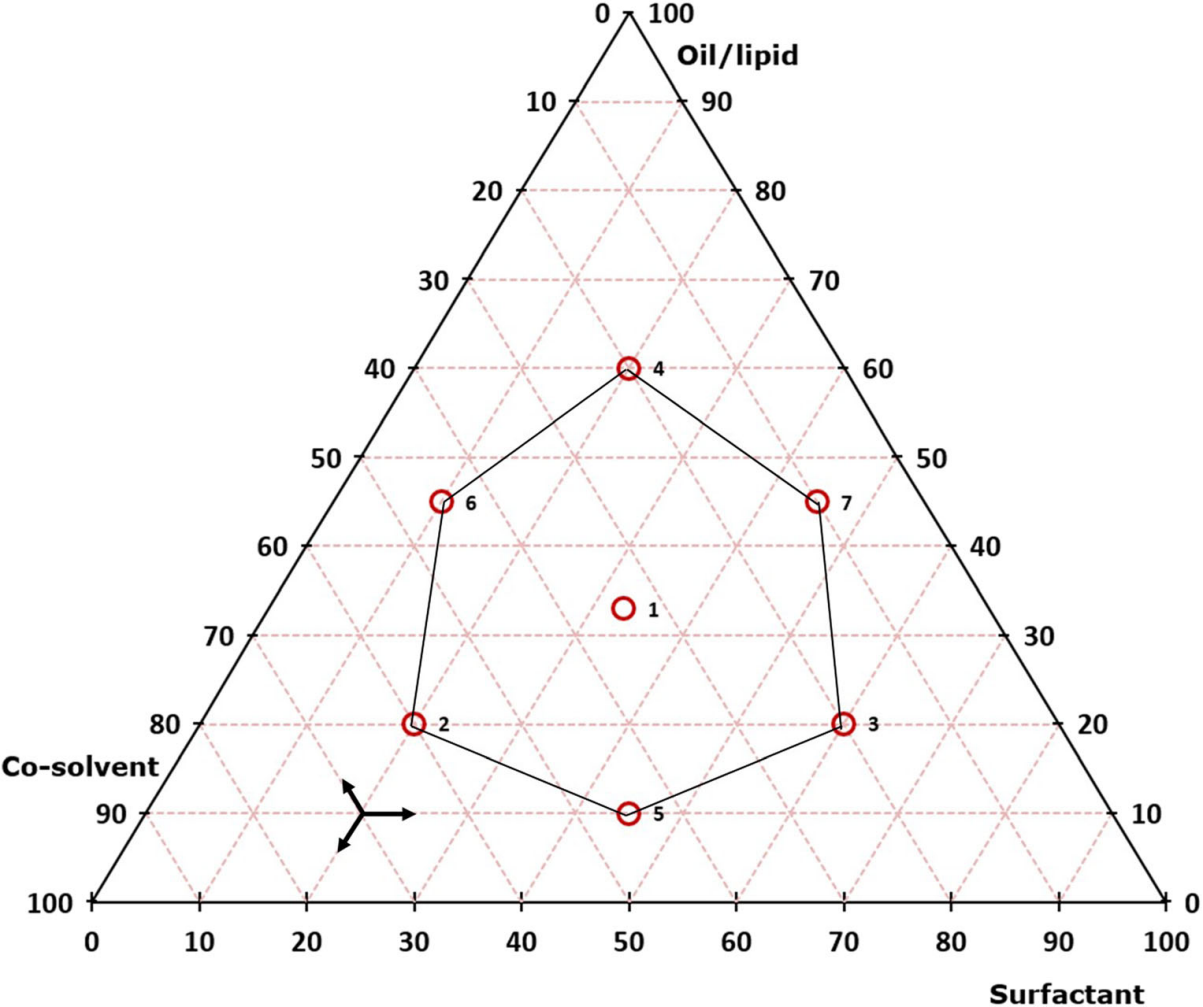
Example for the basic layout of the triangular mixture design applied in determining optimal SNEDDS mixtures for celecoxib and fenofibrate

Equation (1) contains a drug-specific factor *f* to ensure that the drug is completely dissolved in the mixture of the individual components even at varying quantity ratios, the determined saturation solubility *c*_*s*_ (in mg/g) of the drug in the individual components, and the number of individual components *n* used for the SNEDDS formulation. Experiments were performed as follows: in both cases, as a first step, a mixture comprising equal portions of all components, represented by the center point of the triangle, was prepared (sample 1). Then, a hexagon-shaped mixture region around this center point was mapped (Fig. 1), whereas only those mixtures described by the vertices of the hexagon were prepared and analyzed (samples 2–7). With the information obtained from the analyzed SNEDDS mixtures, attention for further formulation optimization was focused on the so-called designated area of interest (DAOI) (*i.e.*, the area within the triangular mixture design that provided SNEDDS that were closest to the above self-imposed specifications). For this step, other geometric figures such as parallelograms and trapezoids were placed into the DAOI, and the SNEDDS mixtures described by the vertices (parallelogram) or the vertices plus one or two more mixing ratios along the parallel sides (trapezoid) of these geometric structures were analyzed (samples 8–11 or 8–14, respectively) until a SNEDDS formulation meeting all specifications was identified.

### Emulsification Performance (Visual Observation)

To ensure that SNEDDS mixtures capable of forming a stable and translucent nanoemulsion after dispersion in gastrointestinal fluids are determined, emulsification performance tests were performed as follows: a volume of 300 µl SNEDDS containing 25 mg of the specific drug was added to 500 ml water in a 600-ml low type borosilicate glass beaker. The mixture was stirred on a magnetic stirrer for 120 min at 37 ± 0.5 °C. A stirring speed of 150 rpm was set to ensure sufficient mixing, but to prevent vortex formation. All emulsification experiments were conducted using the same settings. Whether a translucent nanoemulsion had formed was visually assessed within the first 5 min of these 120 min. Visual assessment of the emulsions was performed using a grading system, with grade I representing excellent emulsifying properties and grade V indicating that no emulsion had formed (29). All SNEDDS mixtures that did not provide a nanoemulsion after dispersion were discarded. All nanoemulsions were tested for their stability over 120 min. The appearance of turbidity and precipitation of the drug substance and/or the excipients within these 120 min also led to the rejection of the respective SNEDDS mixture. All samples that provided a stable nanoemulsion after dispersion in water were retested for emulsification performance in 0.1 N hydrochloric acid using the same procedure.

### Droplet Size Analysis and Zeta Potential

Besides visual inspection, samples obtained in the emulsification experiments with water as the emulsification medium were subjected to investigation of droplet size distribution by dynamic light scattering (DLS) technology and surface charge analysis (zeta potential) using a Zetasizer Nano ZS (Malvern Analytical Ltd., Malvern, UK). For these experiments, an aliquot of 1 ml was withdrawn after 10 min of stirring as described in the section “Emulsification Performance” and analyzed for droplet size and size distribution.

### Transmittance Measurement

The transmittance of the samples was analyzed to identify stable nanoemulsions. A high transmittance (> 99%) indicated promising sample candidates with a small droplet size, a low chance for drug precipitation, and the potential for a high drug release. Like for the droplet size analysis, after stirring aqueous SNEDDS dispersions in the emulsification performance tests for 10 min, another aliquot (150.0 µl) was removed for transmittance measurement. The aliquot was transferred to a polystyrene 96-well plate (Cellstar, Greiner Bio-One, Kremsmuenster, Austria), and transmittance was measured at 650 nm by UV/Vis spectroscopy using a multiplate reader (Tecan Infinite M200 Pro, TECAN Group Ltd., Männedorf, Switzerland) using purified water as a blank (24, 30, 31).

### Proof of Concept via Statistical Analysis

For proving the concept of the customized mapping method in a triangular mixture design, a statistical analysis of the values linked to droplet size, transmittance and emulsification performance of the analyzed celecoxib and fenofibrate SNEDDS samples was performed using Umetrics software MODDE 9.1.1 from MKS Instruments AB (Malmö, Sweden). Coefficient plots, scatter plots, and contour plots were established to investigate the impacts of varying the SNEDDS composition according to the scheme shown in Figs. 3 and 4 on droplet size, transmittance, and emulsification. As a final, statistical analysis, which essentially required the identification of statistically significant terms, main terms (*e.g.*, "Mig") and alternating terms (*e.g.*, "Mig*PS8") were implemented for the dataset of fenofibrate SNEDDS and for celecoxib SNEDDS; also, quadratic terms (*e.g.*, "Mig*Mig") were implemented to identify statistically significant trends in a coefficient diagram. In the established coefficient diagram, a significant term is represented by a large distance to the line *y* = 0 and an uncertainty level that does not cross *y* = 0. By contrast, a non-significant model term is a model term close to the line *y* = 0 and with an uncertainty level that extends beyond *y* = 0. The statistical analysis was extended by applying observed *vs*. predicted scatter plots to demonstrate the goodness of fit of the collected data. A high *R*^2^ value indicates a good correlation, where ideally all sample points are very close to a regression diagonal.

Finally, contour plots were used to visualize the three-dimensional datasets for celecoxib and fenofibrate SNEDDS in two-dimensional plots for droplet size, transmittance, and emulsification grade. The range of all values of interest (droplet size in nm, transmittance in %, and emulsification grade according to Singh et al*.*(29)) was divided into a specific number of subranges by contour lines, and each subrange was assigned a color. The color assignment across the triangle geometry was used to visualize the values for droplet size, transmittance, and emulsification grade at each point of the triangle, whereby each individual point in the diagram can be assigned to a defined mixing ratio of the excipients used. In the present case, the target range for droplet size (< 50 nm) and emulsification grade (I) is in the purple area of the respective diagrams, and that for transmittance in the orange area of the related diagram.

### Encapsulation Efficiency

To determine the encapsulation efficiency of the selected celecoxib and fenofibrate SNEDDS, a quantity of 1.0 g of each of the corresponding SNEDDS formulations was used. SNEDDS samples were transferred to a small safe-lock tube and centrifuged for 1 min at 9300 rcf and defined quantities of the supernatants (~ 25.0 mg, exactly weighed) were transferred into a 50-ml volumetric flask. Then, first, to each SNEDDS sample was added 10–15 ml mobile phase described in the sections “HPLC Method for Analyzing Celecoxib” and “HPLC Method for Analyzing Fenofibrate”, and the mixture was subjected to ultrasonic treatment for 5 min. Then, each sample was filled up to a total volume of 50.0 ml with mobile phase, thoroughly mixed and analyzed via HPLC. Finally, encapsulation efficiency for celecoxib and fenofibrate was calculated based on the experimentally determined drug load and the theoretical drug load of the corresponding celecoxib and fenofibrate SNEDDS.

### Dissolution Studies with Drug-Loaded SNEDDS

Dissolution experiments were performed with selected celecoxib and fenofibrate SNEDDS formulations. All experiments were performed in triplicate with 25 mg of the specific drug, or an equivalent amount of drug-loaded SNEDDS (300 µl) using USP apparatus II (DT 800 LH, ERWEKA GmbH, Langen, Germany). Dissolution was studied in 0.1 N hydrochloric acid (pH 1.0), acetate buffer USP (pH 4.5), and phosphate buffer USP (pH 6.8). All experiments were performed in a media volume of 500 ml at 37 ± 0.5 °C using a paddle speed of 100 rpm. All samples were withdrawn automatically using a fraction collector, equipped with cannula filters of 10 µm pore size and manually diluted 1:1 (v/v) with acetonitrile before HPLC analysis.

### Stability Studies

Quantities of 10 g each of the selected celecoxib and fenofibrate SNEDDS samples were placed into a 30-ml amber glass jar which was closed with a screw cap and stored at constant and controlled conditions (30 °C/65% RH) in a climatic chamber from Binder GmbH (Tuttlingen, Germany) for 3 months. After 3 months, aliquots from each sample were removed and again studied for encapsulation efficiency, dissolution performance, as well as droplet size, PDI, and surface charge (zeta potential) after dispersion. Results from these studies were compared with those obtained immediately after manufacture.

## RESULTS

### Solubility Data

**Fig. 2:**
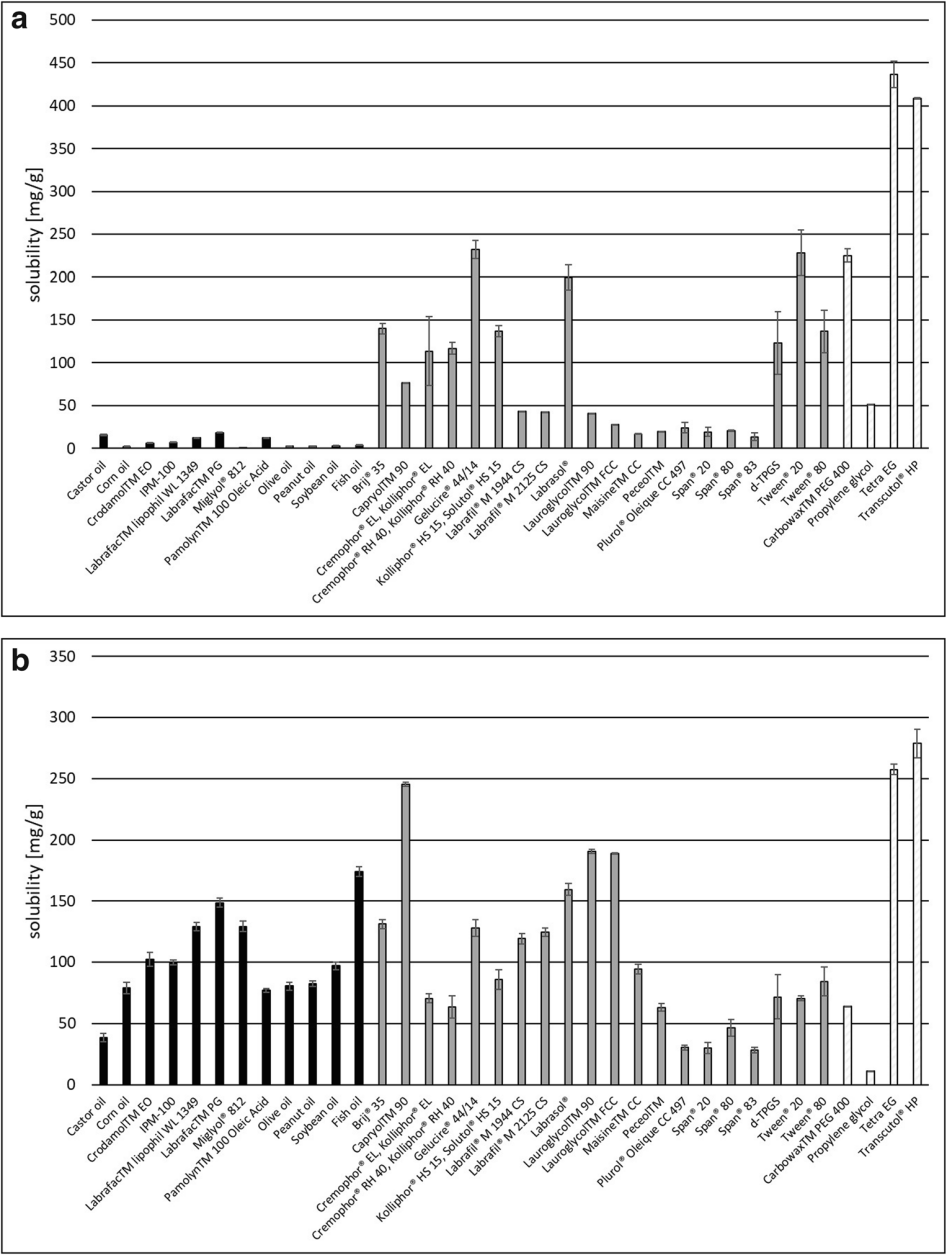
Solubility of celecoxib **a** and fenofibrate **b** in various excipients. Each value designates the mean ± S.D. of *n* = 3. The oils/lipids are colored black, the surfactants grey, and the co-solvents light grey with diagonal stripes

The solubility data (expressed as weight percent) of the model drug substances in a selection of excipients are depicted in Fig. 2a and  b. The excipients are sorted according to their function highlighted by different colors in the bar chart.

### SNEDDS Formulation Design

Excipient mixtures were selected based on the solubility of the drug substance in the individual excipients and the compatibility of the excipients with each other and with the drug substance. The compatibility referred to the miscibility of the excipients with each other and the drug. Another selection criterion was the emulsification performance of the drug–excipient mixture in water. For celecoxib, an excipient mixture of Miglyol^®^ 812, Gelucire^®^ 44/14, Tween^®^ 80, and d-TPGS was selected for further development. Even though celecoxib showed a high solubility in some of the co-solvents (*e.g.*, Tetra EG, Transcutol^®^ HP, Carbowax™ PEG 400) (Fig. 2a), the emulsification properties of these candidates were inappropriate when combined with several oily components and surfactants. Therefore, they were not considered for SNEDDS formulation. By contrast, Miglyol^®^ 812, which revealed exceptionally low solubility for the drug substance celecoxib, was quite easy to emulsify with the surfactant mixture selected in the prescreening experiments and thus, regardless of the solubility data, used for SNEDDS development. For both model drugs assessed in the present study, the combination of Tween^®^ 80 and d-TPGS in a mixture ratio of 8:1 (w/w) or 5:1 (w/w) respectively, represented a well-working surfactant mixture for SNEDDS development. These two excipients were thus part of all formulations and two more compounds, selected from oils/lipids, surfactants, or a surfactant–co-solvent mixture, were added. Fenofibrate SNEDDS consisting of Miglyol^®^ 812, Brij^®^ 35, Tween^®^ 80, and d-TPGS were selected for further assessment. In several other surfactants (*e.g.*, Capryol™ 90, Labrasol^®^, Lauroglycol™ 90, Lauroglycol™ FCC), an increased solubility of the fenofibrate (Fig. 2b) had been observed, but the respective surfactants were not suitable for establishing a stable nanoemulsion. Similar observations were made for the co-solvents Transcutol^®^ HP and Tetra EG, which also presented with a high solubility of fenofibrate, but were not able to establish a stable nanoemulsion and thus not considered in further formulation steps. The compositions of the final SNEDDS formulations for each of the drug compounds are depicted in Table I.

**Table I:** Final Composition of SNEDDS Formulations Incorporating Celecoxib and Fenofibrate

Drug-SNEDDS formulation	Compound 1 (%)	Compound 2 (%)	Compound 3 (%)	Compound 4 (%)	Drug substance (%)
Celecoxib SNEDDS	Miglyol^®^ 812	Tween^®^ 80	Gelucire^®^ 44/14	d-TPGS	Celecoxib
	30.27	49.85	4.55	6.24	9.09
Fenofibrate SNEDDS	Miglyol^®^ 812	Brij^®^ 35	Tween^®^ 80	d-TPGS	Fenofibrate
	18.96	9.48	55.29	11.06	5.21

*SNEDDS*, self-nanoemulsifying drug delivery system

### Individual Designs of the SNEDDS Optimization Approach and Proof of Concept Via Statistical Analysis

**Fig. 3:**
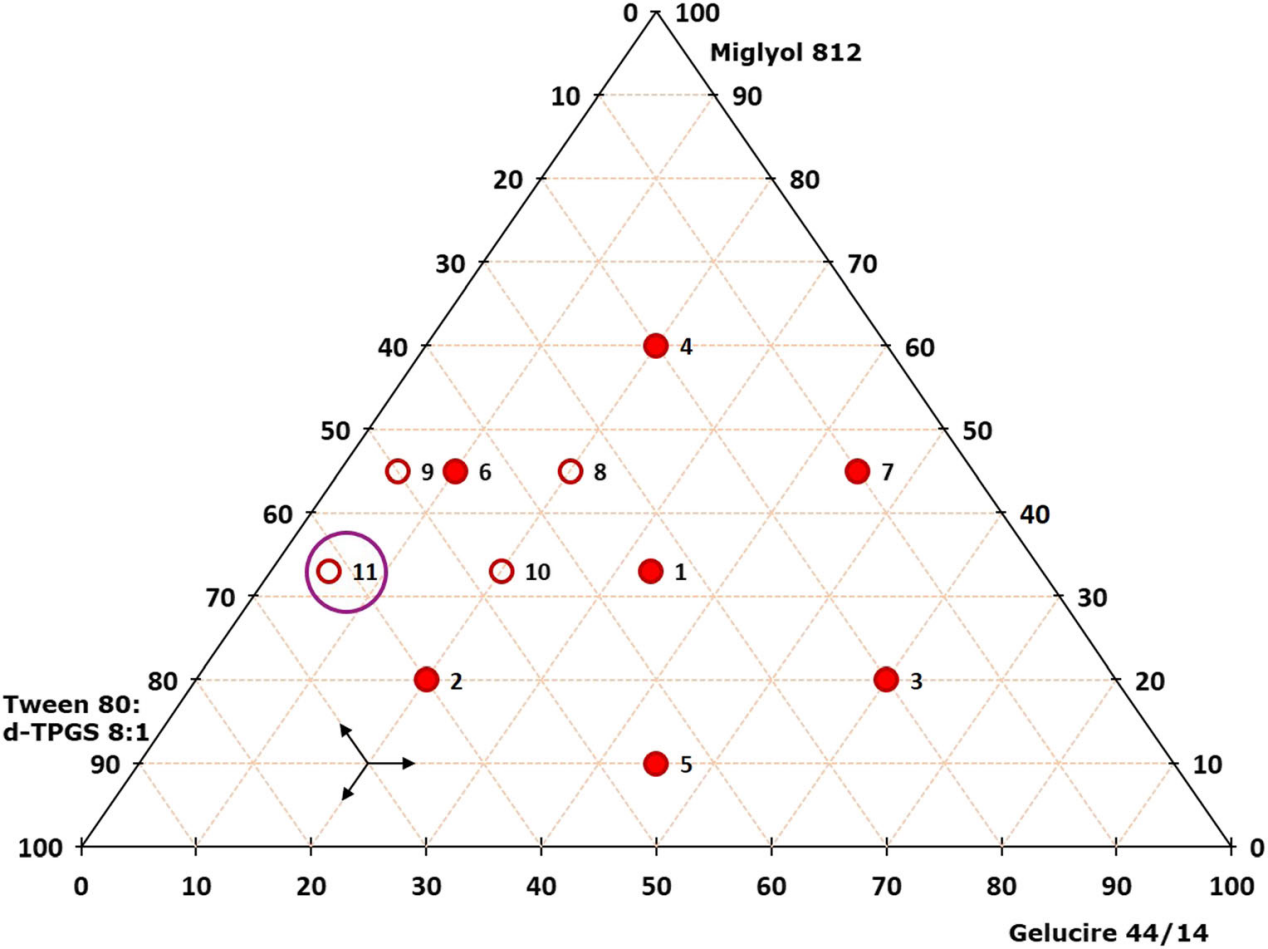
Triangular mixture design applied for the SNEDDS mixture optimization for celecoxib. The filled symbols (symbols numbered 1 to 7) demonstrate the basic structure including the center point and the surrounding hexagon shape. The unfilled symbols (symbols numbered 8 to 11) show a parallelogram structure for further optimization in the DAOI. The circled symbol (symbol numbered 11) represents the final, optimized mixture ratio of excipients for celecoxib SNEDDS

**Fig. 4:**
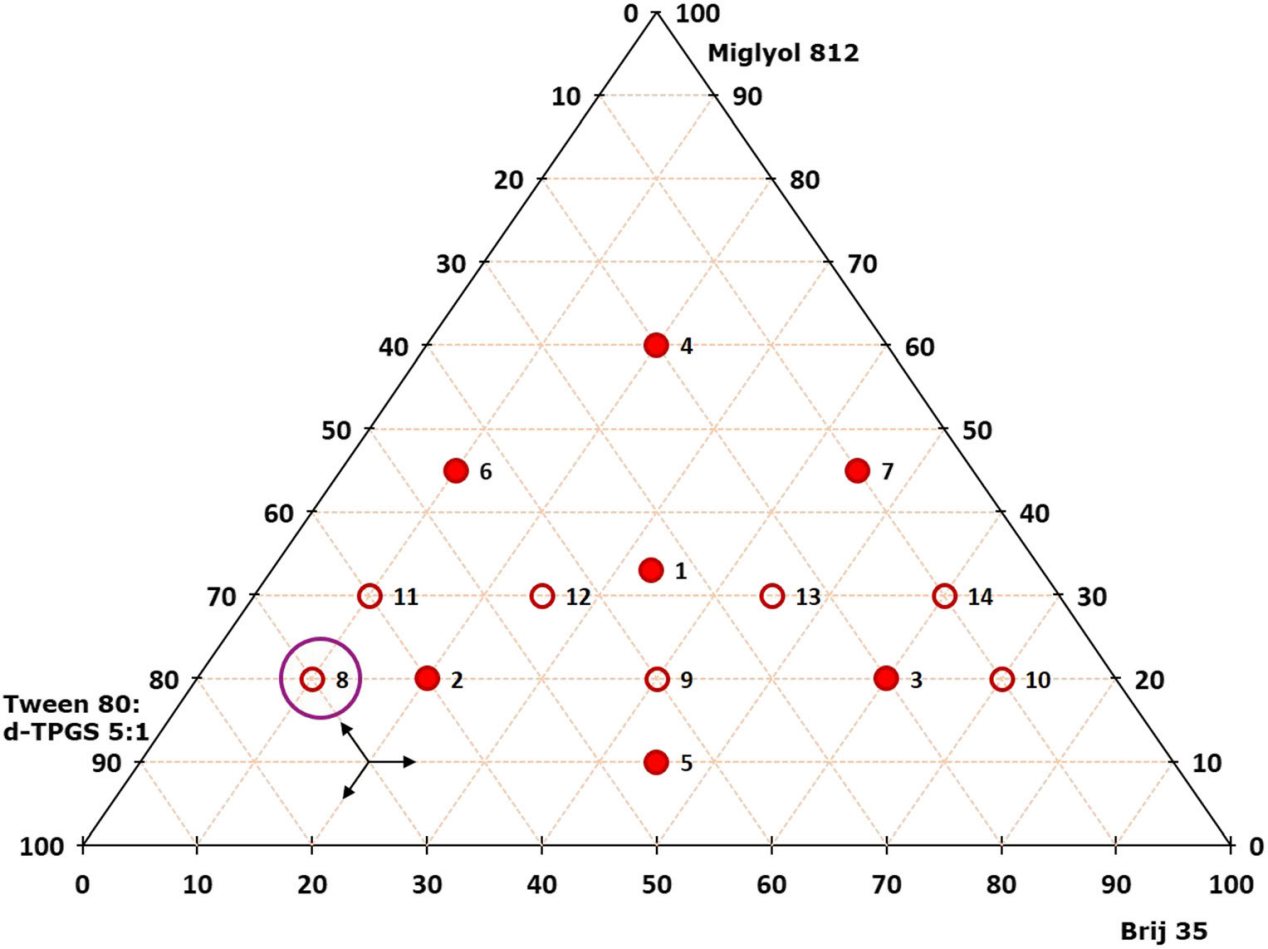
Triangular mixture design applied for the SNEDDS mixture optimization for fenofibrate. The filled symbols (symbols numbered 1 to 7) demonstrate the basic structure including the center point and the surrounding hexagon shape. The unfilled symbols (symbols numbered 8 to 14) show a trapezoid structure for further optimization in the DAOI. The circled symbol (symbol numbered 8) represents the final, optimized mixture ratio of excipients for fenofibrate SNEDDS

The SNEDDS development process using a triangular mixture design targeted to determine a SNEDDS composition for each drug substance that after dispersion provided a droplet size < 50 nm, a PDI < 0.15, and a transmittance > 99%. SNEDDS formulations with different mixing ratios of the selected excipients were prepared. Each formulation exhibited a drug load of 9.09% celecoxib or 5.21% fenofibrate, respectively. The proportional composition of the excipient mixtures analyzed in this initial screening approach can be obtained by reading out the triangular mixture designs provided in Figs. 3 and 4 for each sample by following the arrow directions in the lower left of the graph. Results of the droplet size analysis, PDI, and transmittance of all formulations are shown in Table II. Some mixtures did not form an emulsion after dispersion in water. For each of the two drug substances, results obtained with the majority of the excipient mixtures turned out to be in a reasonable range for the SNEDDS development studies.

**Table II:** Size Average, PDI, and Transmittance of Different Celecoxib and Fenofibrate SNEDDS Formulations Following Dispersion in Water. Sample Numbers Refer to the Corresponding Data Point (Excipient Ratio) Plotted in the Triangle Diagrams (Figs. 3 and 4). Each Value Designates the Mean ± S.D. of *n* = 3

Drug substance	Sample number	Size average (nm) ± S.D.	PDI ± S.D.	Transmittance (%) ± S.D.	Emulsification grade**
Celecoxib	1	58.8 ± 0.5	0.27 ± 0.01	98.9 ± 0.4	II
	2	N/D*	N/D*	N/D*	V
	3	N/D*	N/D*	N/D*	V
	4	150.9 ± 35.1	0.34 ± 0.07	93.5 ± 0.2	IV
	5	N/D*	N/D*	N/D*	V
	6	140.1 ± 41.6	0.20 ± 0.03	99.0 ± 0.1	II
	7	169.2 ± 2.3	0.28 ± 0.02	86.2 ± 0.4	IV
	8	133.3 ± 1.9	0.18 ± 0.01	96.9 ± 0.3	III
	9	83.3 ± 1.6	0.22 ± 0.01	99.2 ± 0.4	II
	10	38.9 ± 0.5	0.23 ± 0.01	99.9 ± 0.4	II
	11	24.4 ± 0.2	0.11 ± 0.01	99.8 ± 0.0	I
Fenofibrate	1	137.5 ± 0.7	0.16 ± 0.01	97.1 ± 0.0	III
	2	34.2 ± 0.2	0.29 ± 0.00	99.8 ± 0.2	I
	3	119.7 ± 1.3	0.20 ± 0.01	98.1 ± 0.2	III
	4	175.6 ± 1.4	0.30 ± 0.00	77.4 ± 0.3	IV
	5	34.7 ± 0.1	0.29 ± 0.00	99.2 ± 0.1	II
	6	143.5 ± 1.3	0.19 ± 0.01	93.6 ± 0.2	III
	7	181.3 ± 3.8	0.23 ± 0.00	87.1 ± 0.6	IV
	8	18.6 ± 0.3	0.06 ± 0.01	99.9 ± 0.1	I
	9	96.3 ± 0.6	0.24 ± 0.01	99.1 ± 0.2	II
	10	114.6 ± 1.2	0.17 ± 0.01	99.1 ± 0.2	III
	11	86.9 ± 0.7	0.19 ± 0.00	99.6 ± 0.1	II
	12	107.4 ± 1.4	0.21 ± 0.01	98.8 ± 0.1	III
	13	156.3 ± 1.5	0.18 ± 0.01	94.6 ± 0.3	III
	14	161.0 ± 2.7	0.17 ± 0.01	92.7 ± 0.7	III

^*^N/D, not determined (composition did not form an emulsion); **assessment according to the grading system by Singh et al. (2008)

When investigating promising excipient mixture ratios for celecoxib (Fig. 3), results from analyzing mixtures 1–7 indicated a DAOI in the vicinity of mixtures 1 and 6 in the triangular mixture diagram. To screen additional mixing ratios in this area for optimizing the SNEDDS composition, a parallelogram providing mixtures 8–11 was inserted for mapping this area in more detail (Fig. 3). Compared with mixtures 1 and 6, the mixtures 10 and 11 showed the desired results in terms of the droplet size and the transmittance, while mixture 11 also showed a considerably lower PDI. The best performing SNEDDS formulation for celecoxib obtained by applying this initial screening approach was thus mixture 11, which after dispersion presented itself as a stable nanoemulsion with the smallest droplet size and the lowest PDI and met all self-imposed specifications. In determining the best SNEDDS compositions for fenofibrate, results from analyzing mixtures 1–7 indicated a DAOI in the vicinity of mixtures 1, 2, 3, and 5. This area was thus mapped in more detail by inserting a trapezoid providing mixtures 8–14 (Fig. 4). In contrast to mixtures 2 and 5 which met the requirements for droplet size and transmittance, most of these additional mixtures did not meet with any of the three specifications. By contrast, mixture 8 which was in close vicinity to mixture 2 provided the best results meeting all specifications.

**Fig. 5:**
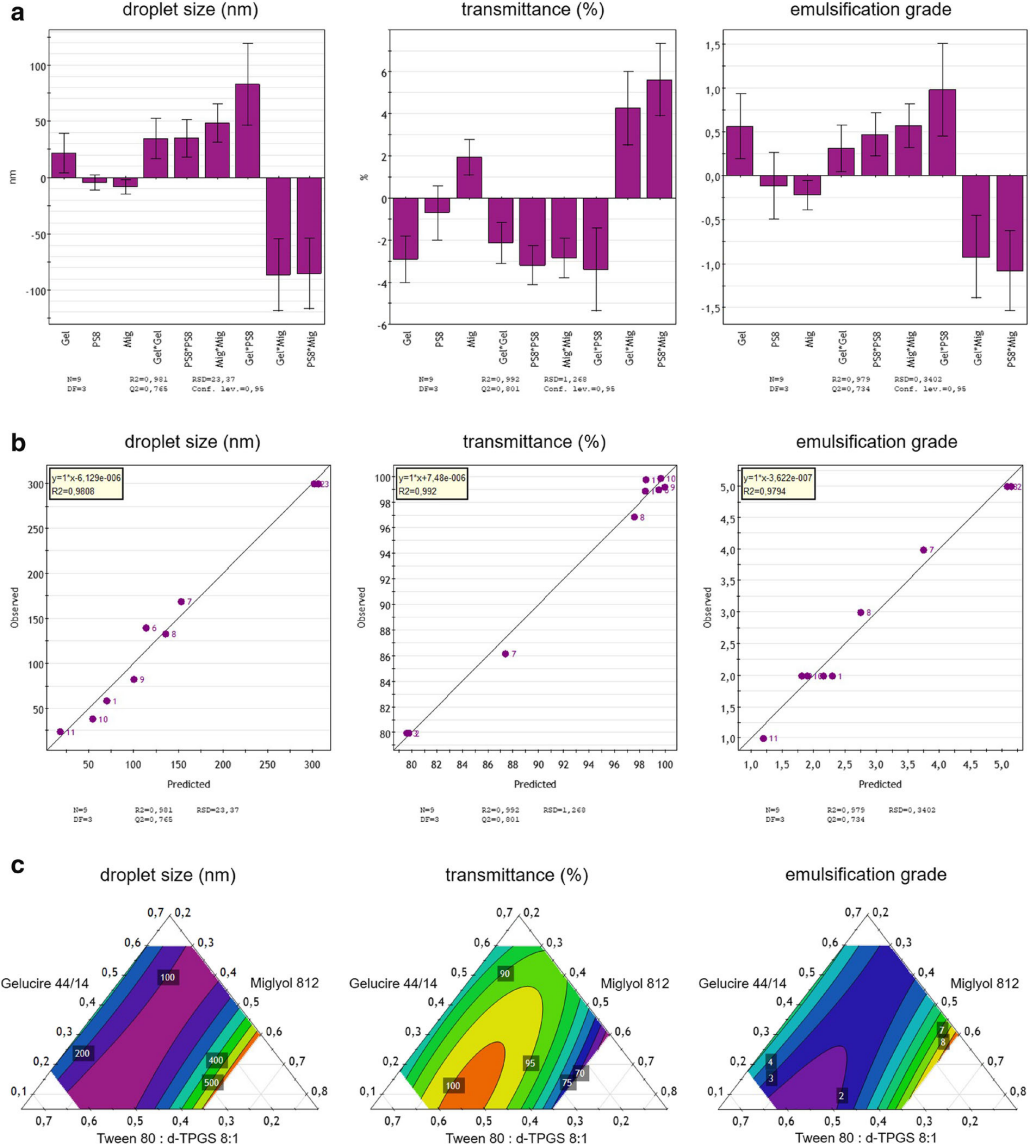
Statistical modelling of the analyzed celecoxib SNEDDS samples applying a coefficient plot **a** (“Gel” = Gelucire® 44/14, “PS8” = Tween^®^ 80: d-TPGS 8:1, “Mig” = Miglyol^®^ 812), an observed *vs*. predicted scatter plot **b**, and a contour plot **c** for the SNEDDS parameters droplet size, transmittance, and emulsification grade

**Fig. 6:**
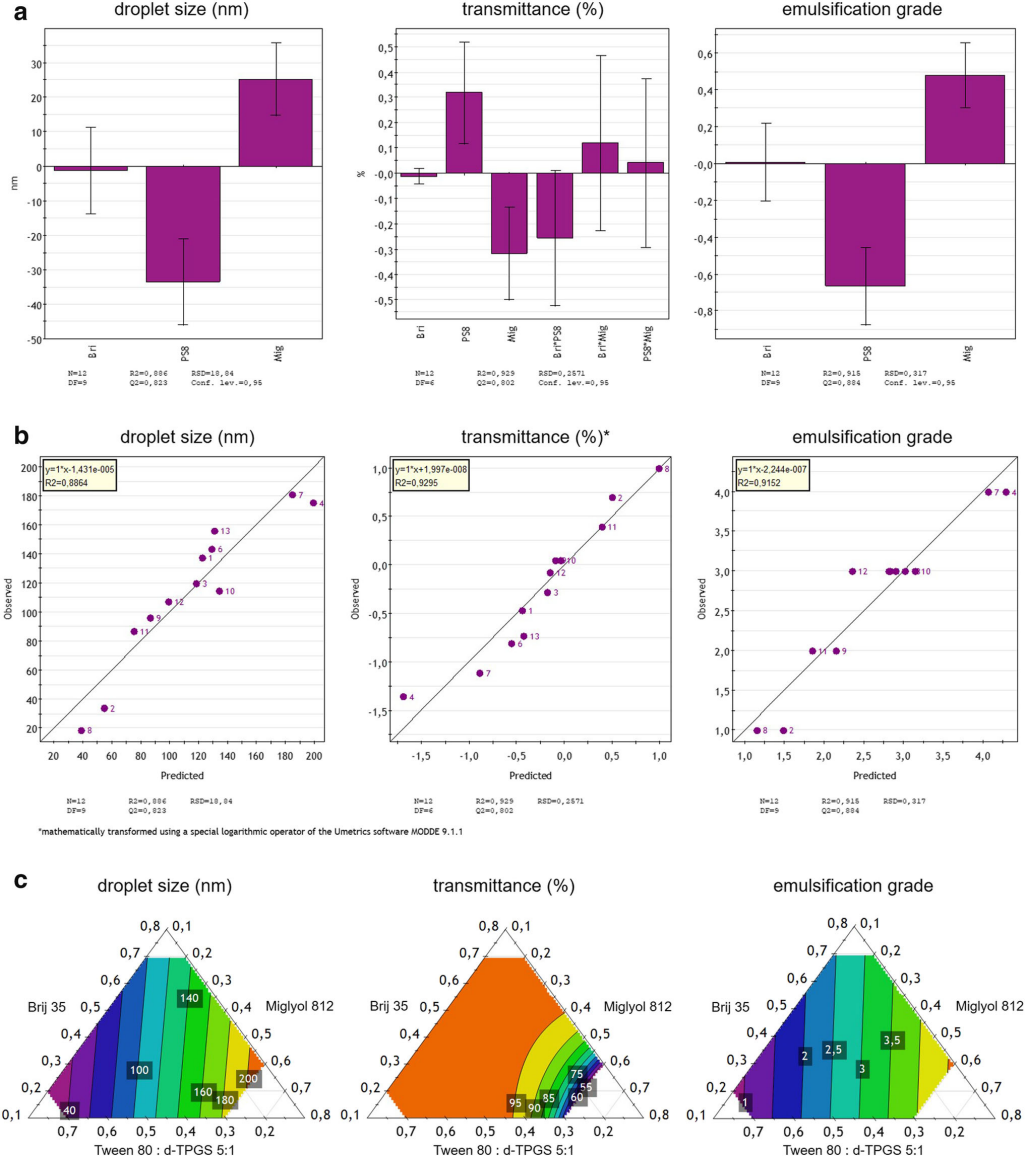
Statistical modelling of the analyzed fenofibrate SNEDDS samples applying a coefficient plot **a** (“Bri” = Brij^®^ 35, “PS8” = Tween^®^ 80: d-TPGS 5:1, “Mig” = Miglyol^®^ 812), an observed *vs.* predicted scatter plot **b**, and a contour plot **c** for the SNEDDS parameters droplet size, transmittance, and emulsification grade

Statistical analysis performed to prove the concept of the tailored screening approach successfully identified significant terms for the celecoxib (Fig. 5a) and fenofibrate SNEDDS (Fig. 6a) datasets using coefficient plots. The celecoxib SNEDDS dataset proved to be more complex in this regard as compared with that of fenofibrate SNEDDS, since implementation of additional quadratic terms was required to determine significant terms for statistical analysis. The scatter plots showed linear regressions for both celecoxib (Fig. 5b) and fenofibrate SNEDDS (Fig. 6b), each with high *R*^2^ values, indicating a sound statistical model. The contour plots (Figs. 5c and 6c) displayed target ranges for droplet size (purple region), transmittance (orange region), as well as for emulsification grade (purple region), which all met the self-imposed specifications for SNEDDS. The contour plot for celecoxib SNEDDS (Fig. 5c) showed that a concentration of Miglyol^®^ 812 between 30 and 40% would be needed to meet the self-imposed specifications for SNEDDS, especially for droplet size and transmittance. The contour plot for fenofibrate SNEDDS (Fig. 6c) indicated that an amount of at least 50% of the Tween^®^ 80: d-TPGS 5:1 blend would be required to achieve the relevant specifications. The mixing ratio of the selected celecoxib (sample 11) and fenofibrate (sample 8) SNEDDS formulations chosen using the new screening tool were each within the statistically determined target range. The statistical analysis thus confirmed the suitability of the tailored screening approach using a specific mapping method in a triangular design for the rapid determination of promising SNEDDS candidates.

### Encapsulation Efficiency

The encapsulation efficiency was > 99% for both celecoxib and fenofibrate SNEDDS (Table III) (*i.e.*, the actual drug load was almost identical to the theoretical one shown in Table I).

**Table III:** Size Average, PDI, and Zeta Potential of Selected SNEDDS Formulations Following Dispersion in Water as well as Encapsulation Efficiency for the Drug Substances Celecoxib (Sample 11) and Fenofibrate (Sample 8) at the Time of Manufacture (0 M) and After 3 Months of Storage at 30 °C/65% RH (3 M). Each Value Designates the Mean ± S.D. of *n* = 3

Sample name	Zeta potential (mV) ± S.D.	Size average (nm) ± S.D.	PDI ± S.D.	Encapsulation efficiency (%) ± S.D.
Celecoxib SNEDDS (0 M)	− 6.62 ± 0.66	24.4 ± 0.2	0.11 ± 0.01	99.98 ± 0.06
Celecoxib SNEDDS (3 M)	− 7.07 ± 0.61	26.1 ± 0.2	0.12 ± 0.01	99.87 ± 0.18
Fenofibrate SNEDDS (0 M)	− 13.10 ± 0.79	18.6 ± 0.3	0.06 ± 0.01	99.90 ± 0.16
Fenofibrate SNEDDS (3 M)	− 9.34 ± 1.72	21.7 ± 0.2	0.11 ± 0.01	99.40 ± 0.13

*SNEDDS*, self-nanoemulsifying drug delivery system

### Dissolution Performance of Drug-Loaded SNEDDS

**Fig. 7:**
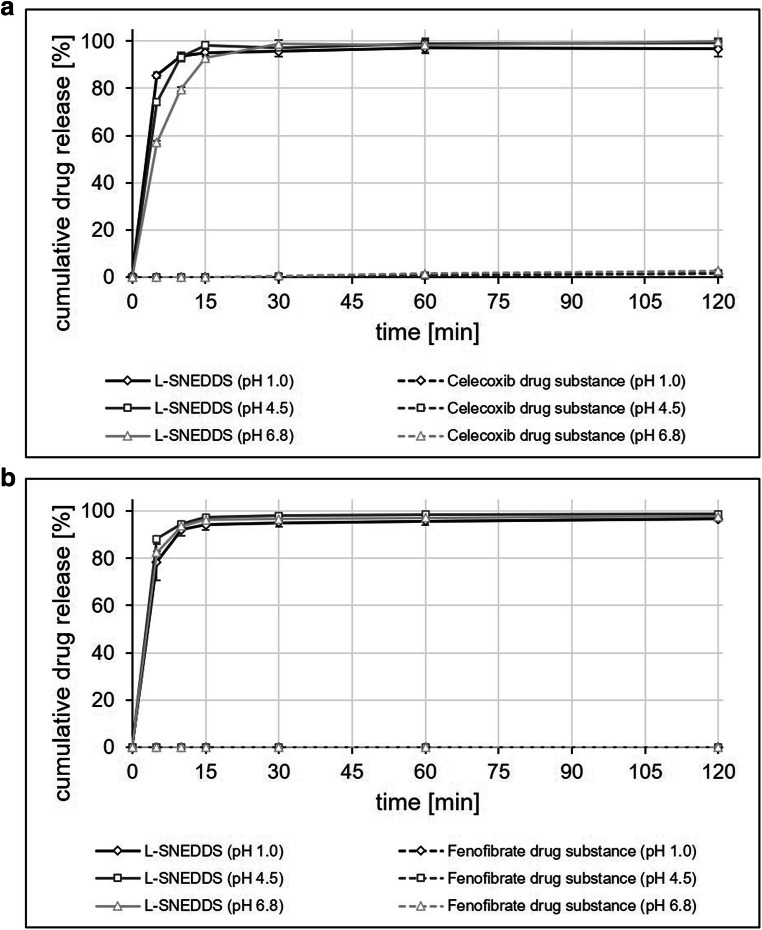
Drug release profiles of celecoxib SNEDDS **a**, fenofibrate SNEDDS **b**, and the corresponding drug substances at the time of manufacture in 500 ml of 0.1 N hydrochloric acid (pH 1.0), acetate buffer USP (pH 4.5), and phosphate buffer USP (pH 6.8) using USP apparatus II at 100 rpm. Each value designates the mean ± S.D. of *n* = 3

Whereas the dissolution experiments confirmed the poor solubility of the two model drugs, both celecoxib and fenofibrate SNEDDS formulations showed fast and complete dissolution in media of pH 1, 4.5, and 6.8, and no precipitation was observed over the test duration of 120 min. (Fig. 7a–b).

### Stability Studies

For the celecoxib SNEDDS, all results obtained in the 3-month stability study at 30 °C/65% RH were similar to those obtained immediately after manufacture (Table III) (*i.e.*, encapsulation efficiency, as well as droplet size, PDI and surface charge (zeta potential) after dispersion did barely change. Results for fenofibrate SNEDDS were mostly similar but presented with a slight decrease of the surface charge (zeta potential) and a slight increase of the PDI of the droplets after dispersion (Table III).

**Fig. 8:**
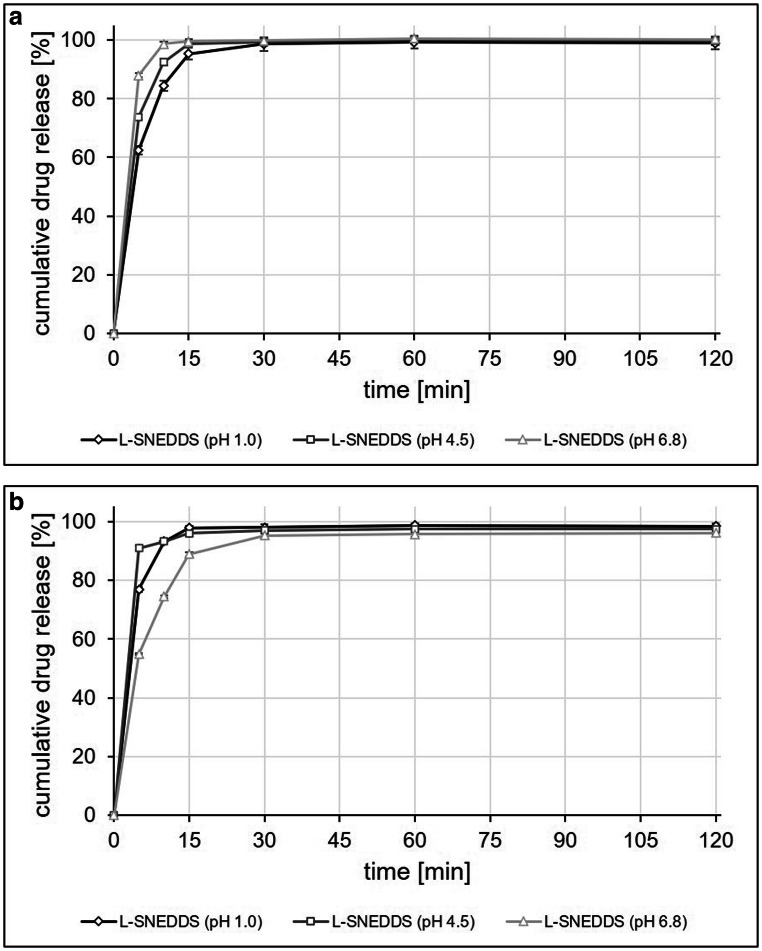
Drug release profiles of celecoxib SNEDDS **a**, fenofibrate SNEDDS **b**, and the corresponding drug substances after 3 months of storage in 500 ml of 0.1 N hydrochloric acid (pH 1.0), acetate buffer USP (pH 4.5), and phosphate buffer USP (pH 6.8) using USP apparatus II at 100 rpm. Each value designates the mean ± S.D. of *n* = 3

Storage conditions did not have an impact on the dissolution performance of both celecoxib and fenofibrate SNEDDS. As observed in the initial set of dissolution experiments, drug release was fast and complete with > 90% of the dose released within the first 15 min of the experiment in all media (Figs. 7a–b and 8a–b), and no drug precipitation was observed over the duration (120 min) of the experiments.

## DISCUSSION

In this study, a special triangular mixture design was applied for determining appropriate excipient ratios for SNEDDS formulations of two poorly water-soluble drug compounds. The presented development and optimization approach for SNEDDS distinct from the conventional 10% increment procedure offered the opportunity to substantially diminish the number of trials from about 50 to 11 or 14 individual experiments, respectively. Based on reducing the number of screening experiments, this approach is very efficient in saving time and resources. Several analytical methods were strategically combined to determine the best SNEDDS composition for a given drug compound. Each analytical method on its separate basis provided limited utility for SNEDDS development, but the combination of selected analytical results provided information on key properties, particularly emulsification performance, droplet size, PDI, and transmittance after dispersing the drug-loaded SNEDDS in water. The target drug loads for the SNEDDS formulations were calculated based on Eq. (1). A drug-specific factor *f* was applied to circumvent a limitation due to the saturation solubility of the drug in the excipient mixtures, especially when varying the excipient quantity ratios, since in the concentration range close to the saturation solubility of the drug in the excipient mixture, the risk of generating unstable emulsions that fail in the emulsification performance evaluation and then could not be further utilized was likely to be high. By considering the varying excipient mixture ratios as regarded from the center point of the mapping method in the triangular mixture design, value 0.7 was chosen as drug-specific factor *f* for celecoxib SNEDDS and 0.5 for fenofibrate SNEDDS. As a result of the initial observations in the emulsification performance experiments, a smaller value for the factor *f* was applied for fenofibrate than in the case of celecoxib to ensure that both celecoxib and fenofibrate SNEDDS formulations formed stable emulsions. However, three SNEDDS formulations of celecoxib did not provide stable emulsions as shown in Table II so that the information level for the mapping method in the triangular design was limited for the development of celecoxib SNEDDS. Since these three SNEDDS formulations did not provide stable emulsions for the statistical evaluation, theoretical worst-case assumptions regarding droplet size, transmittance, and emulsification grade were made for these samples to enable establishment of a statistical model. Moreover, in the applied model, results for two samples for each drug substance (samples 4 and 5 for celecoxib SNEDDS and samples 5 and 14 for fenofibrate SNEDDS) were identified as statistical outliers and therefore excluded from the statistical analysis.

Analytical parameters obtained for the best performing SNEDDS formulations were compared with literature data from various SNEDDS studies. The droplet size of the final SNEDDS composition containing celecoxib was much smaller than the range of the droplet sizes achieved by Song et al*.*(32), Shaji et al*.*(33), and Salem et al*.*(2). Moreover, both the obtained droplet size and the PDI of celecoxib SNEDDS were smaller than those reported by Chavan et al*.*(19) and Yakushiji et al*.*(34). The final fenofibrate SNEDDS formulation revealed both a smaller droplet size and a lower PDI than the SNEDDS formulations developed by Mohsin et al*.*(12), Eleftheriadis et al*.*(35), Tran et al*.*(36), Bahloul et al*.*(37), and Alshamsan et al*.*(25). All results were characterized by low standard deviations indicating the robustness of the methodology. Compared with the cited studies on SNEDDS development for the active ingredients celecoxib and fenofibrate, the approach using a special triangular mixture design provided formulations that might exhibit a better *in vivo* performance. Administering SNEDDS that provide much smaller droplet sizes after dispersion may lead to better drug absorption associated with increased bioavailability (14, 38). A smaller droplet size also provided an indication of better physical stability (15) over the time as revealed by the emulsification performance tests. Enhanced drug release and absorption as well as increased bioavailability from smaller droplet sizes of two otherwise identical formulations have been demonstrated for emulsions (39) and also for self-emulsifying drug delivery systems (38, 40, 41), although the influence of lipid digestion should not be neglected (41). The slight increase in droplet size of the final fenofibrate-SNEDDS formulation may be related to the slightly decreasing zeta potential of the formulation during the storage period, as a higher zeta potential generally indicates a higher stability of the dispersed system. This hypothesis is supported by the fact that the zeta potential for the selected celecoxib-SNEDDS formulation remained almost unchanged over a 3-month storage period, which was also true for the corresponding droplet size of the corresponding formulation.

Results from *in vitro* release experiments with the best performing celecoxib and fenofibrate SNEDDS formulation and the corresponding active ingredients (Fig. 7a–b) clearly demonstrated the impact of formulation properties on extent and rate of drug release. Based on results from the emulsification performance tests, SNEDDS formulations rated grade III (or worse) according to the rating system of Singh et al*.*(29), did not prove to be suitable candidates for drug release studies and were thus not further investigated. The *in vitro* release profiles of the selected SNEDDS formulations shown in Figs. 7 and 8 support the observations made in the emulsification performance tests. The celecoxib and fenofibrate SNEDDS formulations that immediately after manufacture, but also after 3 months of storage presented with a high encapsulation efficiency, spontaneous emulsification, a small droplet size, and a low PDI (Table III) provided a rapid, complete, and pH-independent drug release for both poorly soluble APIs.

In summary, these data clearly demonstrate the value of the established screening method, which provided a rapid, innovative, and effective (pre-)screening tool approach for the future development of SNEDDS formulations. In order to optimize this screening approach for the development of SNEDDS formulations, which certainly has not yet been fully exploited, consideration could be given to increasing the drug load of the formulations and including a broader range of excipients at the beginning of the screening process. As digestive processes could have a significant impact on the formation of nanoemulsions and therefore theoretically on the bioavailability of the administered SNEDDS formulations, this should be addressed in advanced *in vitro* studies and selected SNEDDS formulations should be finally evaluated *in vivo*.

## CONCLUSION

A novel customized screening approach for rapid SNEDDS development was successfully established and applied to the model drugs celecoxib and fenofibrate. Promising SNEDDS formulations were characterized by a very small droplet size, a low PDI, a high transmittance, and excellent emulsification performance. Results from *in vitro* release experiments indicated a huge increase in both rate and extent of drug release when comparing the performance of celecoxib and fenofibrate SNEDDS formulations with that of the corresponding drug compounds. Overall, results obtained in the study indicate that the novel approach represents a promising platform for efficiently designing stable and rapidly releasing SNEDDS formulations incorporating poorly water-soluble drugs. The approach enabled the rapid determination of optimized SNEDDS formulations with a manageable, limited number of experiments. It could thus streamline the screening process for rapid SNEDDS development and will be further refined in the future.
